# Jet fans in the underground car parking areas and virus transmission

**DOI:** 10.1063/5.0033557

**Published:** 2021-01-12

**Authors:** Ata Nazari, Moharram Jafari, Naser Rezaei, Farzad Taghizadeh-Hesary, Farhad Taghizadeh-Hesary

**Affiliations:** 1 Department of Mechanical Engineering, University of Tabriz, Tabriz, Iran; 2 Department of Clinical Oncology, Shahid Beheshti University of Medical Sciences, Tehran, Iran; 3 Social Science Research Institute, Tokai University, Hiratsuka-shi 259-1292, Kanagawa-ken, Japan

## Abstract

Jet fans are increasingly preferred over traditional ducted systems as a means of ventilating pollutants in large environments such as underground car parks. The spread of severe acute respiratory syndrome coronavirus 2 (SARS-CoV-2)—which causes the novel coronavirus disease—through the jet fans in underground car parks has been considered a matter of key concern. A quantitative understanding of the propagation of respiratory droplets/particles/aerosols containing the virus is important. However, to date, studies have yet to demonstrate viral (e.g., SARS-CoV-2) transmission in underground car parks equipped with jet fans. In this paper, numerical simulation has been performed to assess the effects of jet fans on the spreading of viruses inside underground car parks.

## I. INTRODUCTION

Since December 2019, the novel coronavirus disease (COVID-19) has become a major concern for the global population. It has led to 1 092 144 deaths worldwide, as of October 15, 2020.^1^ It simulated the clinical course of infection with two previously reported human coronaviruses—including severe acute respiratory syndrome coronavirus (SARS-CoV) and Middle East respiratory syndrome coronavirus (MERS-CoV)—which was named severe acute respiratory syndrome coronavirus 2 (SARS-CoV-2) by the Coronavirus Study Group of the International Committee on Taxonomy of Viruses.^2^ Studies have reported that SARS-CoV-2 spreads mainly through respiratory droplets and aerosols.^3–8^ These respiratory droplets can be exhaled during coughing, sneezing, or even talking.^9^

The increase in mortality rates of COVID-19 has prompted scientists to evaluate all aspects of viral transmission. Considering the characteristics of respiratory droplet/particle/aerosol transmission in wind conditions, there will be a large number of viruses within an underground car park when a confirmed case sneezes near the jet fan. Underground car park ventilation will cause cross-infection through respiratory droplet/particle/aerosol transmission among people if the appropriate design is not taken. So far, the underground car park design and viruses’ control have rarely been studied directly.

The jet fan ventilation system has been developed to ventilate underground car parks for carbon monoxide (CO) removal during normal conditions as well as smoke extraction in an emergency scenario, such as a fire.^10–13^ Under normal conditions, jet fans can spread the sneeze-originated respiratory droplets in the airflow direction and increase the risk of viral transmission. In this regard, the number of people in underground car parks and jet fans’ velocity play a key role in the risk of viral transmission.

Comprehensive reviews of COVID-19 transmission via respiratory droplets were conducted by Carelli,^14^ Drossinos and Stilianakis,^15^ Chen,^16^ and Chen *et al*.^17^ Sun and Zhai^18^ analyzed the infection probabilities of COVID-19 via large respiratory droplets and recognized 1.6 m–3.0 m as a safe social distance. Dbouk and Drikakis^19^ numerically studied airborne droplet transmission during coughing. They found that respiratory droplets could travel unexpected considerable distances depending on the high-speed wind conditions. Dbouk and Drikakis^20^ also demonstrated that respiratory droplets from coughing or sneezing traveled a distance less than 2 m in the case of zero-wind conditions. Bourouiba^21^ found that expelled respiratory droplets during human sneezing could travel up to 7 m–8 m at 36 km/h–108 km/h wind speeds. Various researchers recommended the use of face masks in the public environment, and some of them believed that social distancing of 2 m may not be adequate during the COVID-19 outbreak.^22^

Suspended respiratory particles—originated from cough or sneeze—^23–25^ will severely influence the air quality in hospitals/health care,^5,26,27^ schools,^28^ airplanes,^29,30^ and various closed environments.^31–33^ In the COVID-19 pandemic, suitable heating, ventilation, and air-conditioning (HVAC) systems may have a completing role in mitigating the potential airborne transmission of SARS-CoV-2. Chaudhuri *et al*.^34^ performed an analytical study on the respiratory droplets’ role in the COVID-19 pandemic. They derived the infection rate constant by respiratory droplet collision rate theory. Busco *et al.*^35^ proposed a novel technique to predict the spread of aerosol and droplets accurately. De-Leon and Pederiva^36^ demonstrated a kinetic Monte Carlo algorithm for modeling different scenarios of the SARS-CoV-2 infection rate. Cummins *et al.*^37^ investigated the effects of gravity on various-sized respiratory droplets. They found that gravity has an essential role in the modeling of sneezing or coughing so that in the absence of gravity, the behavior for the droplets is not uniform. Dbouk and Drikakis^38^ introduced a new Eulerian–Lagrangian multiphase computational fluid dynamics (CFD) solver based on theoretical correlations for the transient effects on respiratory droplets’ heat and mass transfer. Mittal *et al.*^39^ presented a mathematical model for estimating the risk of SARS-CoV-2 transmission. They demonstrated that the increase in physical activity/exercise might increase the transmission risk. Smith *et al.*^40^ modeled the dynamics of exhaled respiratory droplets to account for the aerosol persistence times in confined public environments. Fontes *et al.*^41^ presented the numerical analysis of the effect of human physiology factors on the respiratory droplet transmission of SARS-CoV-2. They showed that an ill host may be less likely to transmit a pathogen when they frequently blow their nose.

All the above-mentioned modeling approaches and experimental visualizations are valuable and may be suitable for future medical and engineering analyses. Recently, two comprehensive studies of the SARS-CoV-2 behavior were studied by Kanso *et al.*^42^ and Chen *et al.*^43^ Kanso *et al.*^42^ developed a new and interesting method to study SARS-CoV-2 virus behavior. Their work was based on sculpting the coronavirus particle from tiny beads and then applying the laws of fluid physics to each bead. In addition, they calculated the properties of SARS-CoV-2 from its shape. Chen *et al.*^43^ demonstrated that treatments with near-room-temperature, cold atmospheric plasma can kill SARS-CoV-2 present on a variety of surfaces in as less as 30 s.

Shang and Xing^44^ compared two induced ventilation systems in an underground garage with different ways of air exhaust. The two air exhaust ways were upper exhaust and upper 1/3 and lower 2/3. Their results showed that the air distribution of the 1/3 upper and 2/3 lower exhaust systems is better. The jet fan that is close to air exhaust had an important role in exiting the pollutant (i.e., CO) from the underground garage. By dividing the exhaust duct into two parts, the final jet fan push pollutants move to the outlet easily with lower pollutant concentration near the ducts.

Li and Xiang^45^ investigated metal particulate pollution in the underground car park with various mass concentrations. Their system ventilation did not use the jet fan. They showed that wetting the road surface reduces the concentration of metal particulate pollution remarkably. They stated that this finding is due to decreasing the suspension of soil dust.

Viegas^46^ realized that when the jet fan flow rate in an underground car park is smaller than the exhaust flow rate, recirculating flows increase and disperse pollutants. This lack of emergency scenario can improve visibility but may increase the average pollutant (i.e., CO_2_) concentration. Špiljar *et al.*^11^ showed that increasing the number of jet fans does not improve the mechanical ventilation system efficiency. The selection of the number of jet fans, the distance between them, and the power of the extraction fans should be simulated by computational fluid dynamic software.^47^ Kmecová *et al.*^48^ proposed the important point of the design during the fire scenario inside the underground car park. Results elaborated that exhaust shafts should not be located in both parts of the car parking.

Infection control and prevention depend on disrupting the human-to-human transmission of pathogens (in this case, viruses). Understanding routes of disease transmission and how it contributes to the spread of viruses allows for the identification of effective prevention and control measures. The transmission of viruses can be divided into the following five main routes: direct contact, fomites, aerosol or airborne, oral route, and vector-borne. Surprisingly, the SARS-CoV-2 pathogens may be transmissible through unexpected daily activities, for example, the turbulent flow induced by toilet flushing,^49^ the male-oriented urinals,^50^ and the exhausted aerosol from the clean-room heating and ventilation conditioning systems.^5^ Transmission via each of these three routes is important and depends on various factors (e.g., the particle distribution, the turbulent intensity, the humidity, and the temperature). Among these, SARS-CoV-2 mainly has an airborne transmission potential.^51^ Continuous airflow of jet fans can increase the transmissibility of virus-containing droplets/particles/aerosols in underground car parks. A better understanding of how respiratory droplets/particles/aerosols spread due to the continuous airflow of jet fans may provide insight that contributes to mitigating SARS-CoV-2. To the best of our knowledge, the problem of spreading viruses in underground car parks has not been investigated previously. All previous works of the literature focused on CO control. The main aim of our work is to present predictions of the respiratory droplet/particle/aerosol spreading, resulting from the continuous airflow of jet fans. This helps investigators gain a deep understanding of the behavior of complex air flows inside underground car parks, which are engaged with the dynamics of viruses. The simulations were carried out at a low-speed of jet fans (daily applications or normal condition) with various configurations of the sneeze source. Finally, we suggest six tips to reduce the risk of infection through the respiratory droplet transmission during the use of the jet fan air-conditioning system in underground car parks.

## II. JET FANS WITHIN THE UNDERGROUND CAR PARKING AREAS

In recent years, the growing concern over the poor air quality inside underground car parks has accelerated the research studies in the field of heating, ventilating, and air conditioning (HVAC) significantly. Great efforts have been carried out by researchers to improve the air quality of underground car park areas.^52,53^ Jet fans and ducted systems have been introduced as a means of ventilating pollutants from underground car park areas. Even if both techniques help to remove the pollutants, they are significantly different. The jet fan ventilation offers advantages over ducted ventilation for underground car parks:^54–57^

• No ducting in the parking area, reducing fan pressure, kW, Specific Fan Power (SFP) (energy savings).
• More space for parking, improved visibility and appearance (space-saving, less dead zones).
• May reduce the height of parking space, saving building cost (flexible installation, cost savings).
• Possibility for smoke control systems.

On the other hand,

• Ducting is prone to damage and obstructs other services.
• Ducting needs cleaning and maintenance.

Underground car park ventilation jet fan systems can be designed for three objectives in the event of a fire (emergency scenario):

• Assist fire-fighters to clear smoke from an underground car park area both during and after a fire outbreak.
• Provide clear smoke-free access for fire-fighters to a point close to the seat of the fire.
• Protect the means of escape from the car park.

The discharged velocity of jet fans differs between day-to-day ventilation (regarding CO and virus-containing particles) and smoke extraction ventilation in case of an emergency scenario. Jet fans’ speed typically had two orders of low velocity (day-to-day ventilation) and high velocity (emergency scenario). These configurations and discharged velocity of jet fans depend on the architecture of an underground car park area.

CO and nitrogen dioxide (NO_2_) are the most relevant air pollutants inside underground car park areas. In general, petrol engine vehicles (mainly cars) are the source of most but not all CO in car parks, and diesel engine vehicles are the source of most but not all NO_2_. CO blocks the absorption of oxygen by the blood, and this can lead to dizziness, unconsciousness, or death depending on the concentration. NO_2_ affects the lungs and may cause breathing difficulties, prompt asthma attacks, and induce long term damage to the lungs. To provide adequate protection of public health, the air quality inside car parks should be kept within the ranges mentioned in Table I. A case study of underground car park geometric particulars is demonstrated in Table II. The thrust force of each jet fan is 27 N and 50 N for day-to-day and fire mode scenarios, respectively. The Reynolds number of each jet fan is in the range of 70 000 to 200 000 equal to the velocity of 5 m/s–14 m/s, and flow discharge contains the turbulent air flow of the free jet.

**TABLE I.** The allowable carbon monoxide level in underground car parking areas.

Organization	Ventilation rate	PPM
ASHRAE 62-1999^54^	7.5 L/s m^2^	25
ASHRAE 62-1999^54^	7.5 L/s m^2^	35
U.K standard^57^	6-10 ACH	50
U.K standard^57^	6-10 ACH	90

**TABLE II.** Underground car park geometric particulars.

Title	Value
Maximum length	122 m
Maximum width	86 m
Net parking area	5978 m^2^
Height	2.9 m
Number of fire zone	3
Number of jet fans	20

The jet fan ventilating system—in underground car parks—had advantages against the ducted system, but this system can spread respiratory particles too far away from the source of sneeze or cough. We focus on this concern and propose several solutions.

## III. KEY ISSUE OF JET FAN AIR-CONDITIONING SYSTEMS WITHIN THE UNDERGROUND CAR PARKS IN THE COVID-19 SITUATION

**FIG. 1.**
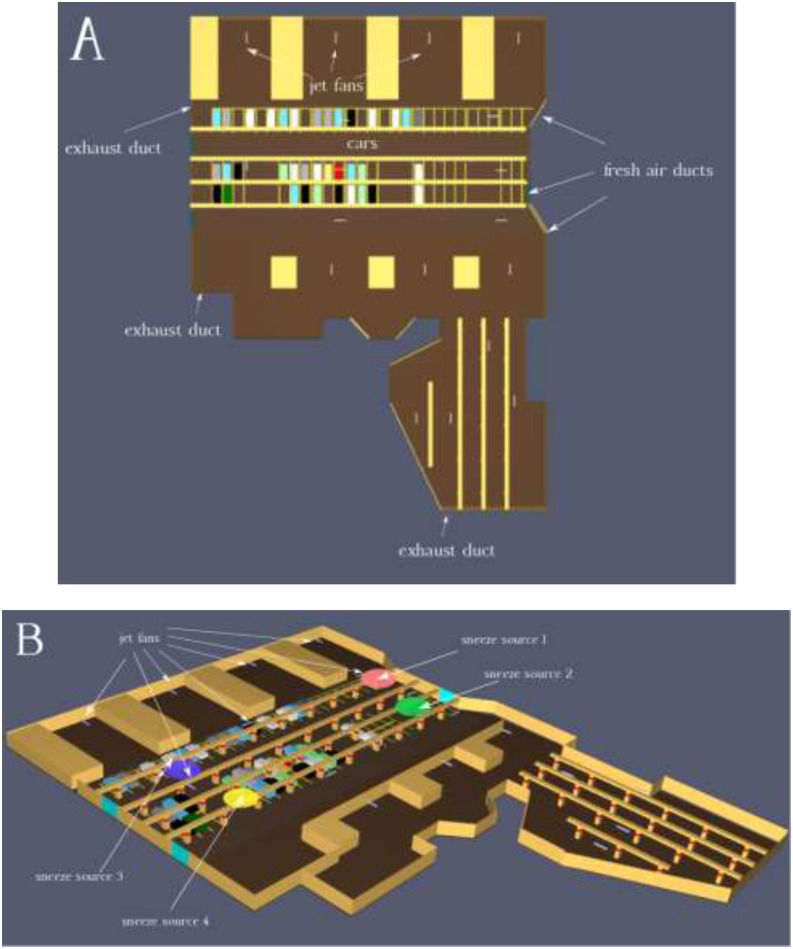
Schematic view of underground car parks containing fresh air ducts, exhaust ducts, jet fans, and cars (a) and locations of the sneeze sources (b).

**FIG. 2.**
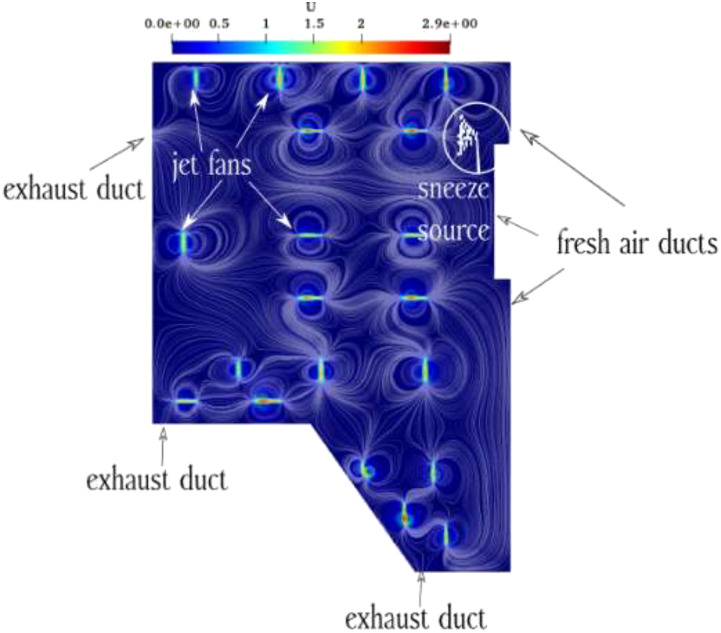
Schematic view of the created streamline due to the continuous jet fan air flows and sneeze sources.

The ventilation system in underground car parks consists of jet fans, fresh air ducts and exhaust ducts, CO detection sensors, and a control panel.^48^ This system—which operates in a similar way to a ducted system—is based on placing a set of axial impulse fans all along the underground parking area. When the jet fan conditioning system is installed on the ceiling, it moves the air toward the exhaust ducts by effectively creating a continuous flow and impeding the creation of stagnant zones. Therefore, the jet fan system—with the continuous flow—can easily spread the virus-containing droplets/particles/aerosols inside underground car parks. For example, when an infected person sneezes near the jet fan, the respiratory droplets/aerosols spread through the jet fan system. Figure 1 represents the schematic view of the computational domain, including the fresh air ducts, exhaust ducts, and the configuration of the jet fans, and we assumed four different locations as the sneeze sources inside underground car parks. The spreading patterns of the viral particles after a human cough or sneeze near the jet fan are demonstrated in Fig. 2.

## IV. FORMULATION OF THE JET FAN FLOWS IN THE UNDERGROUND CAR PARKS

The ventilation process of underground car parks involves the continuous incompressible form of the air, in a process of jet fan fluid flow. To track the viral dynamics, we applied the Reynolds-Averaged Navier–Stokes (RANS) method for the modeling of interactions between jet fan fluid flows and created respiratory particles. The jet fan flow turbulent effects modeled using the Reynolds-averaged form of Eqs. (1)–(3) are called RANS equations along with the standard *k* − *ω* turbulence model. In addition, the transmission of respiratory droplets/particles/aerosols containing the virus under the action of jet fans is tracked by using the discrete phase model (DPM), which is a Lagrangian tracking approach.^58^

### A. RANS model

The relevant equations of motion are the continuity equation, the momentum conservation law, and temperature equation in their incompressible form, respectively, represented as follows:^59^

$$\frac{\partial }{\partial x_{j}}\left(\rho u_{i}\right)=0,$$ (1)

$$\begin{array}{ll} & \frac{\partial }{\partial t}\left(\rho u_{i}\right)+\frac{\partial }{\partial x_{j}}\left(\rho u_{i}u_{j}\right)\\ & \;=\frac{\partial p}{\partial x_{i}}+\frac{\partial }{\partial x_{j}}\left[\mu \left(\frac{\partial u_{i}}{\partial x_{j}}+\frac{\partial u_{j}}{\partial x_{i}}\right)\right]+\frac{\partial }{\partial x_{j}}\left(-\rho u_{i}^{\prime }u_{j}^{\prime }\right), \end{array}$$ (2)

$$\rho C\frac{DT}{Dt}=\nabla \cdot \left(k\nabla T\right)+\frac{1}{2}\tau :\left(\nabla u_{i}+\nabla u_{j}^{T}\right),$$ (3)

where *p*, u, and T are the pressure, the fluid velocity, and the temperature of fluid, respectively.

### B. Discrete phase model (DPM)

The DPM is adopted in this paper to simulate the virus-containing particle movement under the effect of continuous airflow of jet fans in underground car parks. Recently, Dbouk and Drikakis,^19^ Li *et al.*,^49^ and Wang *et al.*^50^ used this model to simulate a human cough-induced particle movement, turbulent induced toilet flushing, and urinal transmissions, respectively. The flow pattern of a particle is calculated from the following equation:^60^

$$\frac{du_{p}}{dt}=F_{D}\left(\vec{u}-{\vec{u}}_{p}\right)+\frac{g\left(\rho_{p}-\rho \right)}{\rho_{p}}+F_{Brownian}+F_{Saf\;fman},$$ (4)

where *d* is the diameter of the sneezed respiratory droplets/particles carrying viruses between 1 *μ*m and 13 *μ*m; the resulted Stokes drag force equation is adopted to calculate *F*_*D*_ as follows:^61^

$$F_{D}=\frac{18\mu }{d^{2}\rho_{d}C_{c}},$$ (5)

where the Stokes–Cunningham slip coefficient *C*_*c*_ under atmospheric conditions is calculated to be from the following equation:^62^

$$C_{c}=1+\frac{2\lambda }{d}\left(1.257+0.4e^{-\left(1.1d/2\lambda \right)}\right),$$ (6)

where *λ* is the molecular mean free path of the gas. In addition, because of the size of the respiratory particles, the Brownian force and Saffman lift force are taken as *F*_*Brownian*_ and *F*_*Saffman*_, respectively. The components of the Brownian force are modeled as a Gaussian white noise process with spectral intensity S_n,ij_ given by^63,64^

$$S_{n,ij}=S_{0}\delta_{ij},$$ (7)

where *δ*_*ij*_ is the Kronecker delta function and^64^

$$S_{0}=\frac{216\nu \sigma T}{\pi^{2}\rho d_{p}^{5}{\left(\frac{\rho_{p}}{\rho }\right)}^{2}C_{c}}.$$ (8)

T is the absolute temperature of the fluid, *ν* is the kinematic viscosity, and *σ* = 1.38 × 10^−23^ is the Stefan–Boltzmann constant. Amplitudes of the Brownian force components are of the form^64^

$$F_{Brownian}=\xi_{i}\sqrt{\frac{\pi S_{0}}{\Delta t}},$$ (9)

where *ξ*_*i*_ are zero-mean, unit-variance-independent Gaussian random numbers. The amplitudes of the Brownian force components are evaluated at each time step.

### C. SAFFMAN’S lift force

The Saffman’s lift force can be defined as follows:^64^

$$F_{Saf\;fman}=\frac{2K\nu^{1/2}\rho d_{ij}}{\rho_{p}d_{p}{\left(d_{lk}d_{kl}\right)}^{1/4}}\left(\vec{u}-{\vec{u}}_{p}\right),$$ (10)

where K = 2.594 and d_ij_ is the deformation tensor as well as *d*_*lk*_ and *d*_*kl*_.

## V. NUMERICAL TECHNIQUE AND BOUNDARY CONDITIONS

The open-source field operation and manipulation (OpenFOAM) for computational fluid dynamics (CFD) software package version 5 was used to perform numerical simulations. The OpenFOAM codes were written in the C++ programming language using the finite-volume numerical technique to solve the conservation of mass, energy, and momentum along with the equation of state in their Reynolds-averaged form. The second-order upwind scheme was used to handle the convective terms. The Gauss-linear second-order approach was employed to address the diffusion terms. The Pressure-Implicit with Splitting of Operator (PISO) algorithm was applied to couple the pressure and the velocity. The under-relaxation factors for the pressure, momentum, and energy equations were 0.7, 0.7, and 0.6, respectively. In addition, the minimum residuals for the convergence of pressure, velocity, and temperature were 10^−10^, 10^−11^, and 10^−12^, respectively. In order to simulate the particle movement during the normal condition of jet fans in underground car parks, two assumptions were adopted in this article: (1) the generation of the respiratory particles is ignored, and (2) the size and other physical properties of the respiratory particles remain constant during simulation.

The fixed-value and fixed-flux pressure conditions are imposed at the fresh air ducts. The inlet-outlet are employed to model the exhaust ducts. Our case study can control CO by jet fans in the underground car park without the air circulations. Afterward, we focus on the viruses spreading inside the car park.

**FIG. 3.**
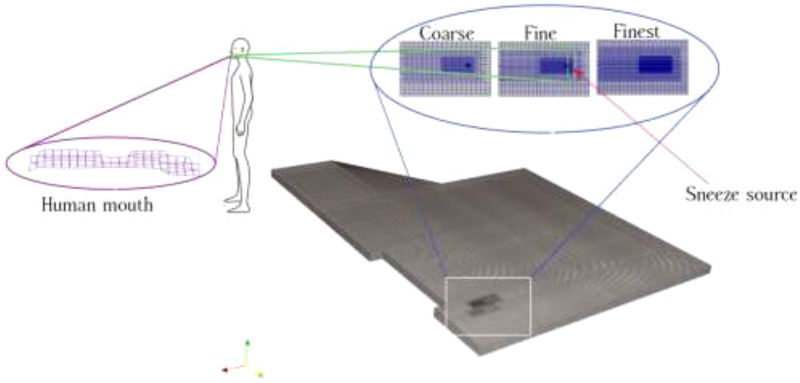
A 3D computational domain grid mesh. The mesh is very refined at the mouth and is gradually coarsened in the stream-wise cough flow direction with multilevel refinement.

Dbouk and Drikakis^19^ conducted experimental measurements during the human cough to capture the effective mouth area during coughing. This method was conducted based on mouth-print quantification via a high-speed camera over 0.12 s. This paper uses Dbouk and Drikakis’s^19^ findings on characteristics of the human mouth opening to determine the effective area of orifice during sneezing/coughing. We demonstrated a 3D computational domain of an underground car park and showed a 2D section of non-uniform structured elements around the sneeze source for coarse, fine, and finest meshes in Fig. 3. To generate an effective computational mesh, we have used the *block-mesh* and *refine-mesh* utilities. The numerical mesh structure was gradually refined from the outer box with an average cell size of 6 × 10^−4^ m toward the mouth by halving the size of the cells. The cell size of the mouth domain was 2 × 10^−6^ m.

The velocity applied at the mouth is 8.5 m/s in the streamwise cough flow direction, as measured by Scharfman *et al.*^65^ and Dbouk and Drikakis^19^*.* Using the mouth hydraulic diameter and the aforementioned velocity, the Reynolds number is *Re* = 4400.

**FIG. 4.**
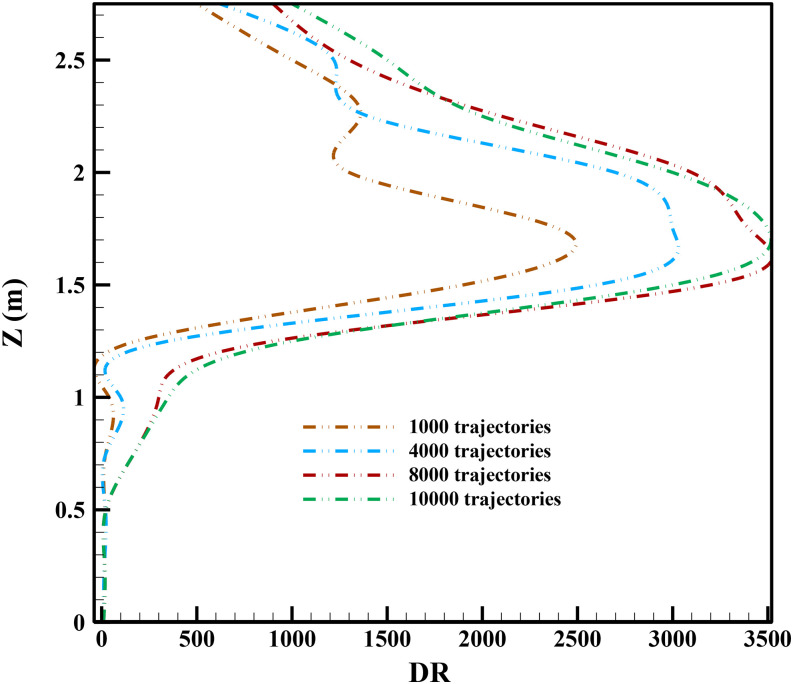
The sensitivity test of the particle number along the ground to roof direction. (DR is defined in Eq. (12) and is the dilution ratio).

Dbouk and Drikakis^19^ stated that by increasing the temperature and decreasing the relative humidity, the evaporation rates of respiratory droplets increase. Released respiratory droplet dimensions during coughing/sneezing are distributed in a wide size range (0.5 *µ*m–1000 *µ*m); most of them quickly evaporate and reach 26% of the initial size (equilibrium diameters) less than 0.3 s in closed environments.^30^ The evaporation process was not considered in our study because underground car parks are a low-humidity environment with relative humidity under 20%,^66^ and the respiratory droplets would form nuclei that mostly encapsulate the virions post-droplet evaporation. The experimental measurements of the relative humidity inside the case study affirm our assumption during the simulation. The average size of particles expelled at the mouth domain is 3.5 *μ*m according to the existing studies by Gupta *et al.*,^67^ Redrow *et al.*,^68^ and Zang *et al.*^69^ To achieve consistent contaminant concentration, Fig. 4 is used. The sufficient number of respiratory droplets/particles based on Fig. 4 is 8000. The x axis of Fig. 4 indicates the sampling direction (Z) from the ground to the roof of the underground car park. Due to the flow direction of jet fans and the spreading of the respiratory along with X and Y directions, we select the Z direction to study the sensitivity test of particle numbers.

## VI. VALIDATION OF RESULTS

**FIG. 5.**
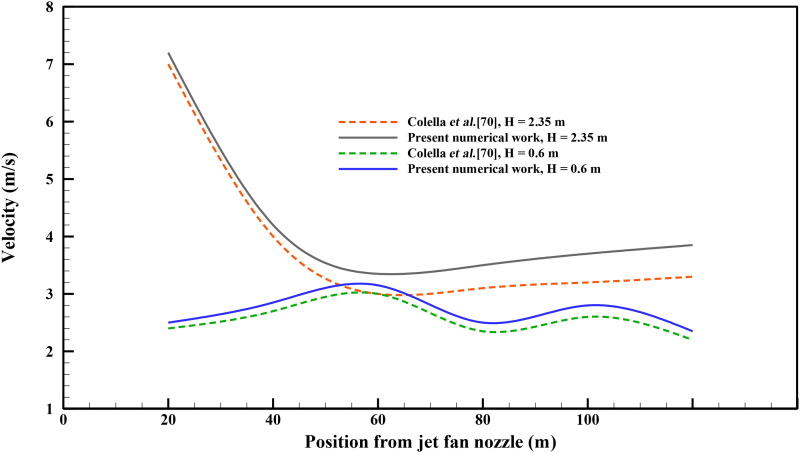
Comparison of the jet fan discharged velocity vs position from the jet fan nozzle with the results of the study of Collela *et al. H* is the height from the ground.

The role of the thrust force in jet fan ventilating systems is vital to push the various pollutants to the exhaust shaft. To ensure proper ventilation inside underground car parking areas, we use the calculated velocity of a jet fan (thrust force) at different positions (sample location). The calculated velocity (thrust force) is determined based on the jet fan fluid core. Figure 5 demonstrates the comparison of our data with the experimental visualizations presented by Colella *et al.*^70^ for the two-dimensional numerical simulation of the discharged velocity for the jet fans. The discharged airflow temperature was set to 20 °C, and the ambient pressure, p_∞,_ was 100 kPa. The maximum relative deviation of the discharged velocity for the two cases in Fig. 5 was ∼4.5%.

**FIG. 6.**
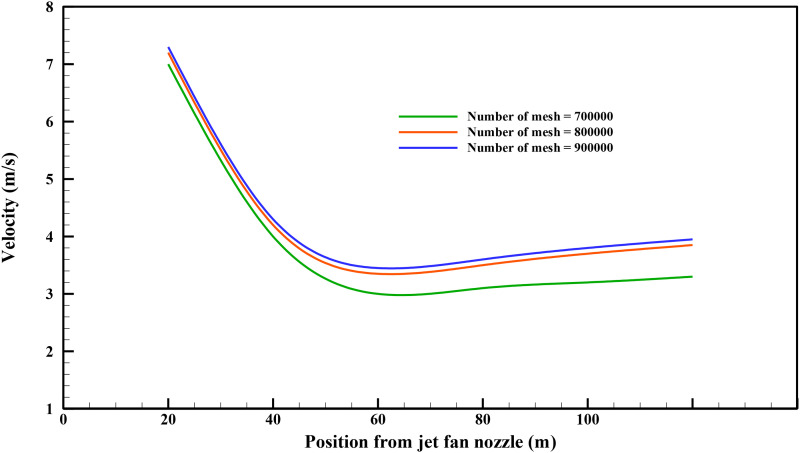
Jet fan discharged velocity vs position from the jet fan nozzle obtained using coarse mesh 700 000, fine mesh 800 000, and finest mesh 900 000 (the structure of these meshes is shown in Fig. 3).

The *grid independence test* was carried out to compute the required number of numerical cells to obtain convergent results. To obtain the grid-independent results, simulations have been carried out on three different mesh topologies at the low-speed jet fan velocity of 10 m/s. Figure 6 presents the jet fan discharged velocity using the three mentioned numerical coarse, fine, and finest meshes. Grids 800 000 and 900 000 produce almost identical results for the jet fan discharged velocity with a relative error of less than 0.5%. Hence, a numerical grid of 700 000 was chosen to have a convergent solution with the optimized computational cost. A summary of the output of the grid independence test is shown in Fig. 6.

## VII. RESULTS AND DISCUSSION

The spreading of respiratory viruses such as SARS-CoV-2 increases when sneeze occurs near the fresh air ducts. The respiratory particle concentrations in underground car parks depend on the jet fan air velocity. Of note, when more than one person exists in the car park, the transmission of virus-containing particles (e.g., SARS-CoV-2) significantly increases. By determining safe and short pathways for exiting attendings in the car park, the risk of transmission will decrease. Clearly, these safe pathways should be near the fresh air and far away from the jet fan flow direction. Currently, the crucial protective effect of the face mask against SARS-CoV-2 has been highlighted by scientific authorities.^71–73^ The persons inside underground car parks should wear the face mask. However, if only one person exists inside the car park, the continuous airflow of jet fans extracts the viral particles from underground car parks, rapidly. Another solution to reduce the viral transmission by the continuous airflow of jet fans is the usage of the high-efficiency particulate air (HEPA) filters and ultraviolet light emitters inside the jet fan boxes. Airflow patterns in underground car parks carry viruses from the sneeze source(s) through the jet fans. Usage of the HEPA filters and ultraviolet light emitters inside jet fan boxes decreases the concentration of viruses.

**FIG. 7.**
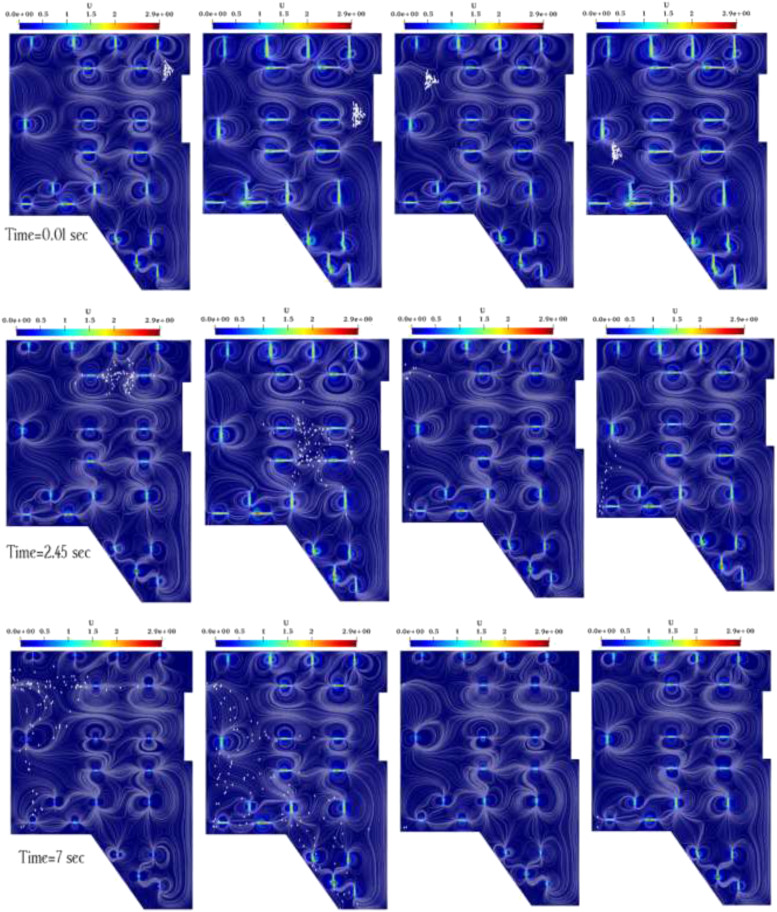
Comparison of the velocity distribution of saliva droplets in underground car parks for sneeze sources 1 (left most column), 2 (middle left column), 3 (middle right column), and 4 (right most column).

Figure 7 indicates the spreading patterns and recirculation of the respiratory particles using the air velocity contours at the four sneeze (infection) sources. Continuous air flow of jet fan transmission is defined as the transmission of infection by sneezed respiratory droplets/particles that are similar to the airborne transmission and can remain suspended in the underground car park for a short time. Over this short time, the discharged particles potentially expose a much higher number of susceptible individuals at a much greater distance from the source of sneeze in the underground car park. By comparing Fig. 7, the distribution of respiratory droplets/particles in the underground car park depends on the location of the sneeze source. Furthermore, the multiple suction and discharge of the respiratory droplets/particles by jet fans convert these particles to the much smaller aerosols that may move further away.

The experimental and numerical data of jet fan velocity field vs position by Colella *et al.*^70^ were firstly selected for model validation. In their study, jet fans’ configuration was built inside an enclosed space to mimic the enclosed environment of underground car parking. The CO distribution and local velocity profiles were measured using the computational fluid dynamic technique. Their measurements from both publication and supplementary materials provided many detailed data for validations. The velocity value measured at the jet fan core zone was selected and compared between the experimental measurements^70^ and our numerical predictions, as illustrated in Fig. 7. The velocity value predicted in this study yielded similar airflow directions and distributions to the experimental results in most of the regions. Given the fact that the dead zone is generally used for emergency conditions, it does not have such application in this study, which aimed at evaluating the distribution of respiratory droplets in the daily mode of car parking ventilation. In the current study, however, all dead zones inside the car parking—where the air velocity is relatively low—are considered as the source of cough/sneeze. Since jet fans facilitate ventilation by suctioning the air, they normally draw a small vortex of fluid flow toward itself. In Fig. 7, therefore, the number of particles in the core of the jet fan is often higher than in other areas, and the flow pattern has a large effect on how the particles are dispersed—considering the parking architecture and the location of the columns.

### A. Definition of safe and unsafe areas based on the infection risk

The day-to-day ventilation rate at the fresh air ducts was set based on the BS.72115 standard.^57^ To imitate the best-case scenario, the maximum air supply of 11 300 cubic feet/min (CFM) per fire zone was considered, which equaled to the air change rate of 10/h at an inlet air temperature of 20 °C. It is worth noting that the fire zone differs from our proposed unsafe zone (viral zone). The fire zone has been designed based on the emergency scenario; on the other hand, the unsafe zone (viral zone) has been defined based on the concentration of respiratory droplets/particles/aerosols and infection risk. Based on the good air change rate at day-to-day applications, the Wells–Riley equation is as follows:^74,75^

$$P=1-e^{\frac{-Iqpt}{Q}},$$ (11)

where P is the probability of infection, I is the number of infection sources, which equals 1 for a single sneeze source, and q is the quantum generation rate by an infected person (quanta/s). For the worst-case scenario of infectious disease transmission, q = a unity infectivity term × number of quanta/unit time, in which susceptible people were assumed to be vulnerable to the pathogen. A unity infectivity term delineates that one quantum is equal to one infectious particle/pathogen,^30^ where p is the pulmonary ventilation rate (m^3^/s), t is the total exposure time (s), and Q is the underground car park ventilation rate (m^3^/s).

The various studies indicate that human movement in closed environments can increase the average infection risk. The average infection risk in closed environments (e.g., underground car park and airplane cabin) depends on the movement behaviors of the individuals and the sneeze source (index patient). In this study, the sneeze source has a constant position for four case studies. To solve the individual movement concern in the underground car park, the circular breathing zone has been defined. According to the Australia Work Safety Standards, some studies defined the breathing zone of each person as a hemisphere of a 300 mm radius.^30^ However, due to the complex/different human movement inside the underground car park, this breathing zone (300 mm radius) was not sufficient. The average human walking speed is about 1.4 m/s, and the radius of the circular breathing zone can be estimated by multiplying this value by exposure time (t). By dividing the whole area of the underground car park on the area of the breathing zone, 285 zones have been determined. Since the exhalation velocity of airflow in the personal breathing zone was small,^30^ the influence of the respiration of sneezing/coughing on cautious jet fan airflow transmission might be considered insignificant. Therefore, only the proposed breathing zones of an individual person are considered as the infection zone. An increase in the concentration of respiratory droplets inside each zone increases the probability of infection.

Another shape of the Wells–Riley equation is defined as^32^

$$P=1-e^{\frac{-qt}{DR}},$$ (12)

where DR is the dilution ratio and its formula is $DR=\frac{C}{C_{0}}$, where *C*_0_ is the respiratory droplet/aerosol/particle concentration exhaled by the sneeze source and *C* is the droplet/aerosol/particle concentration in the underground car park. Shao *et al.* used Eq. (12) to calculate the transmission of viruses in closed environments.

$$P_{tot}=\frac{\sum P_{unsa\;f\;e,i}S_{unsa\;f\;e,i}}{S_{tot}}.$$ (13)

**FIG. 8.**
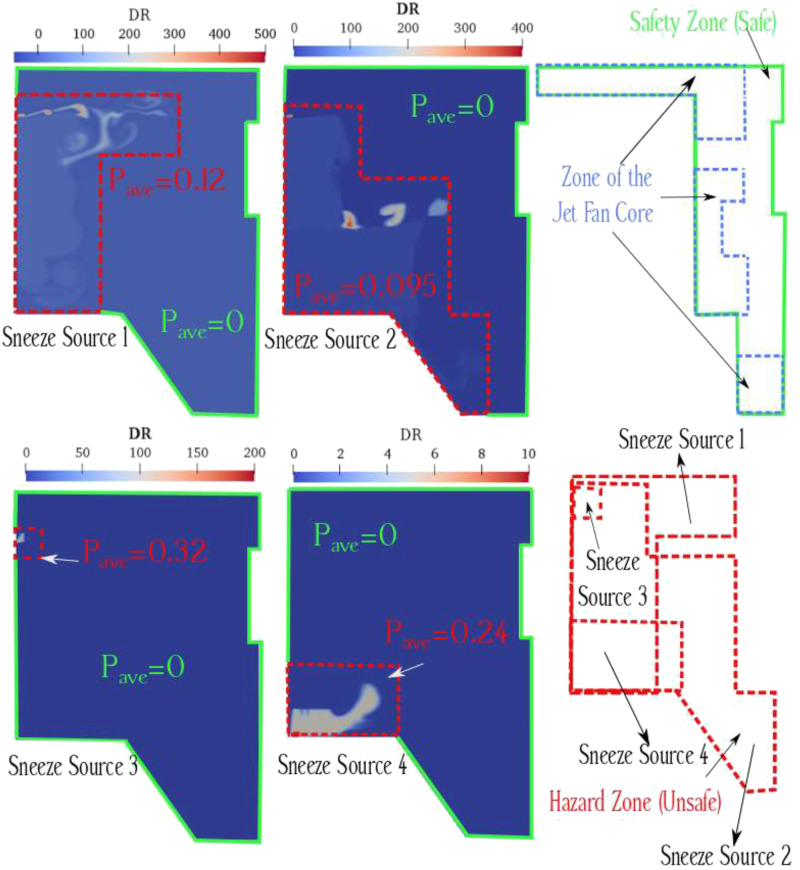
Definition of unsafe and safe zones inside the underground car parking based on the concentration of particles and probability of infection. Zero probability of infection is defined as a safe zone (green lines), and the non-zero area is defined as an unsafe zone (red lines). Due to the definition of the core of the jet flow as an unsafe zone (blue lines), this part has been deducted from the safe zone.

In Sec. VII, we demonstrate that the concentration of particles in the output of each jet fan is higher than in other areas. Using formula (13) and given the proposed respiratory zone, unsafe areas (defined as zones with high particle concentration) for each source of sneezing are calculated. In addition, for each unsafe area inside the car parking, the probability of infection has been calculated (Table III). In general, the probability of infection for the entire parking area is 0.119. Considering the jet fan core as an unsafe area, this area (the blue color in Fig. 8) has been deducted from the safe zone (green areas in Fig. 8)—with zero probability of infection.

**TABLE III.** Values of calculated probability of infection and viral zones area for each case study.

	Area of unsafe	Probability of
Case study	zone (m^2^)	infection (%)
Sneeze source 1	2540	0.12
Sneeze source 2	4101	0.095
Sneeze source 3	45	0.32
Sneeze source 4	720	0.24

**FIG. 9.**
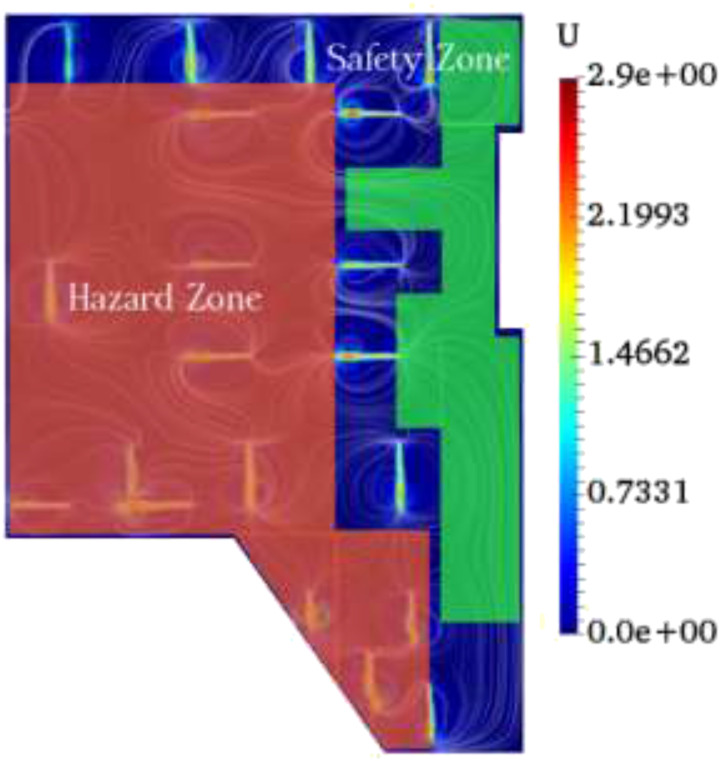
Safety (green) and hazard (red) zones in the underground car park (case study).

**FIG. 10.**
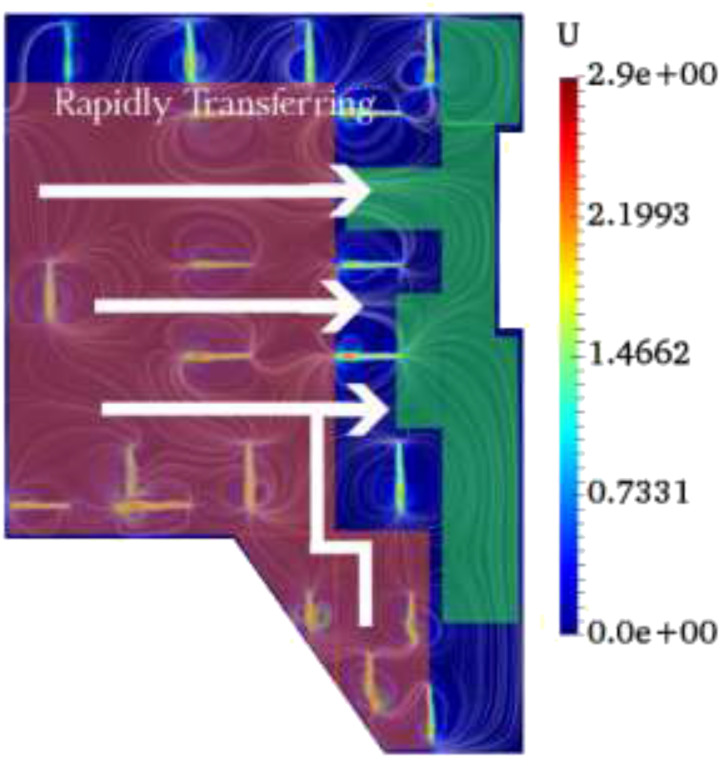
Recommendation on rapidly transferring from the safety zone to the hazard zone in the underground car park.

As demonstrated in Fig. 9, we determined hazard and safety zones based on the concentration of respiratory droplets/particles upon sneeze (as the source of infection). To decrease viral infection (in this regard, COVID-19) transmission, susceptible individuals in underground car parks should try to move in safety zones, as demonstrated in Fig. 10. In the following, we have provided learning tips for traffic in undergrounded car parks, which are ventilated by using the jet fans system:

• to wear a mask,
• to move far away from the core of jet fan flow,
• to leave the hazard zone to the safety zone,
• not to enter the hazard zone for a long time,
• to traffic on lines in the mid-distance of jet fans and parallel to their axis, and
• to find the shortest pathway to exit.

## VIII. CONCLUSIONS

In this article, we computationally investigated the effects of jet fans on the spreading of viruses (e.g., SARS-CoV-2) inside underground car parks. We assumed four different locations as sneeze sources inside underground car parks. The mechanisms of the viruses spreading in a low-speed stream of jet fan air were investigated using the OpenFOAM C++ libraries. After validating the numerical results using the experimental data, several recommendations were offered as follows:

1. Determining safe pathways inside underground car parks will provide greater protection against viral transmission. Due to the jet fan flow directions, these safe pathways should be close to the fresh air ducts.
2. Equipping ultraviolet light emitters and HEPA filters inside the jet fans will also eliminate the viruses.
3. Using face masks is strongly encouraged by authors to prevent the spread of respiratory droplets and aerosols in underground car parks.

## Data Availability

The data that support the findings of this study are available from the corresponding author upon reasonable request.
